# Developing cadres of gender specialists in global public health to meet increasing demand

**DOI:** 10.3389/fsoc.2025.1501764

**Published:** 2025-05-29

**Authors:** Anna Kalbarczyk, Katherine Banchoff, Kelly Perry, Mary de Boer, Rosemary Morgan

**Affiliations:** Johns Hopkins Bloomberg School of Public Health, Baltimore, MD, United States

**Keywords:** gender, training, applied learning, health, global

## Abstract

Gender is increasingly prioritized in global health and embedded across the Sustainable Development Goals. This shift has created a demand for gender specialists with skills to integrate gender into research and programs. Most global health professionals lack formal training in gender analysis or integration. Gender is often misunderstood—conflated with sex, focused only on women, or limited to maternal health. Training can equip professionals to address these gaps, improve outcomes, and reduce inequities. We developed the Gender and Health Summer Institute at Johns Hopkins Bloomberg School of Public Health to grow this capacity. Using universal design, the Institute enhances accessibility, inclusivity, and engagement. Our goal: build a global cadre of gender specialists driving more gender-responsive and equitable health programs.

## Introduction

Gender has been receiving increasing attention in public and global health. For the first time, the global health community has a set of common goals, targets, and indicators that aim to address gender inequality. Not only is gender equality its own sustainable development goal (SDG)—Goal 5: Achieve gender equality and empower all women and girls (United Nations, 2024)—but 14 of the 17 SDGs include related gender equality targets that focus on addressing the social, economic, cultural, and political conditions that reproduce inequality (Odera and Mulusa, 2020). Moreover, the success of Goal 3: Ensure healthy lives and promote well-being for all at all ages, depends on whether gender inequality is addressed.

Gender encompasses the socially constructed roles, norms, responsibilities, and attributes associated with being a man, woman, or gender minority individual in a specific context, as well as the power dynamics between and among these individuals (Darmstadt et al., 2019; Heise et al., 2019). This concept is distinct from, yet related to, gender identity, which refers to an individual's personal sense of having a gender, such as identifying as a woman, man, or gender minority individual. Gender therefore influences how society is organized more broadly, shaping not just individual identities and interpersonal relationships, but also society's norms, institutions, systems, and the resources available for and within families and households, communities, economies and states. Gender also intersects with other social stratifiers, such as race, ethnicity, sexual orientation, age, and disability, to create different experiences of marginalization and privilege (Bose, 2012).

This increased attention to gender is driven, in part, by acknowledgment of the significant negative impacts that gender inequitable health systems can have on health, which manifest in different ways for women, men, and gender minority individuals (Hay et al., 2019; Morgan et al., 2021). Women, for example, have been historically excluded from clinical trials and have faced systematic dismissal or misdiagnosis for their health issues (Merone et al., 2022). Notably, women are less likely to receive pain relief and wait longer to receive it than men with similar symptoms (MacDorman et al., 2021). For women with endometriosis, for example, it typically takes anywhere from four to eleven years between their onset of symptoms and diagnosis (Agarwal et al., 2019; Arruda et al., 2003). Outcomes are often worse for racial and ethnic minority women. Black women in the United States, for example, have up to four times the risk of dying from maternal-related causes than white women, which remains true even when accounting for education and socio-economic status (MacDorman et al., 2021; Berg et al., 2010).

The health of men and boys is also negatively affected by systematized gender inequities (Dworkin et al., 2015). Masculine gender norms are tied to negative or risky health behaviors and low or late care-seeking (Teo et al., 2016; Baker et al., 2014); norms around masculinity can also affect the types of counseling and treatment men receive when they do seek care (Samulowitz et al., 2018). Additionally, providers may exclude or actively restrict men from engagement in maternal or pediatric care provided to their family members, undermining their participation in the health system and health decision-making for and alongside their families (Hay et al., 2019).

Gender inequities also impact the health of gender minority individuals who often face stigma, discrimination, and social exclusion resulting in delayed access to essential healthcare services including gender-affirming care, mental health support and preventive care (Cicero et al., 2019; Turan et al., 2019). These experiences are associated with higher rates of mental health disorders, substance use, and suicide compared to cisgender individuals (Reisner et al., 2016).

Simply put, gender inequity affects everyone. As such, there is an increased need for gender specialists with the skills to integrate gender into programming and research. In response, there has been a growth in the creation of gender advisor jobs in international public health organizations, with many organizations, including UNICEF, WHO, UNDP, and GAVI, valuing gender analysis skills and integration and seeking consultants to conduct gender assessments of projects or contexts. On the job search engine of the United Nations, a search for “gender equality” results in 1,254 vacancies, “gender equity” resulted in 224 vacancies, “GESI” (gender equality and social inclusion) resulted in 131 vacancies, and “gender advisor”, “gender specialist”, and “gender expert” combined resulted in 36 vacancies in June 2024.

However, many health scientists and practitioners are not trained to systematically consider or integrate gender into their research, programs, or practice. There is often a belief that gender integration and analysis can be done by anyone with little to no training, or that gender analysis is as simple as sex disaggregation. Gender is also often conflated with women only or with maternal health. In our own work, we have seen health researchers and professionals argue that anything sex-specific (that is, designed for women or girls specifically) implies a “gender lens”. This is evident in the literature and the ongoing conflation of “sex” and “gender”. Many professionals also struggle to understand what it means to “integrate gender” and to identify key entry points for gender within their research design and methodology. These issues signal a need for applied skills that move beyond a focus on the theoretical.

## Options and implications

Training in gender integration and analysis can prepare researchers and practitioners to meet these new demands and—most crucially—to improve health outcomes and reduce inequities. It is a critical step in creating new paradigms in women's, men's, and gender minority individuals' health. Training, education, and learning on gender integration and analysis helps key actors identify and respond to inequities, as well as to allocate appropriate resources to implement equitable health systems (Morgan et al., 2016). Such training can also help address the lack of awareness amongst researchers and practitioners on the specific health needs and challenges of women, girls, men, boys, and gender minority individuals, as well as encouraging them to engage with their own gender biases and potentially discriminatory attitudes (Oduenyi et al., 2021; Govender and Penn-Kekana, 2008). These trainings enable individuals to conduct gender-intentional research, programming, and monitoring alongside purposefully acknowledging and confronting the ways gender power dynamics intersect with other hierarchies of privilege or oppression, such as race, ethnicity, disability, migration status, religion, sexual orientation, and gender identity.

Despite the need for and benefits of training in gender, gender analysis, and gender integration, there are limited accessible and interactive training opportunities. Existing gender training opportunities for international health professionals include courses offered by Cynara and GenderPro, both led by institutions based in high-income countries (HICs). Cynara courses are offered independently by diverse instructors across a range of topics with costs per course on a sliding scale. The GenderPro Capacity Building and Credentialing Program, hosted at the George Washington University in partnership with UNICEF, aims to educate international development professionals on the best approaches to improve the lives of women and girls around the world. The course is offered over four months twice a year for a cost of $2500. Professionals may apply and be assessed for the credential without having completed the GenderPro Capacity Building program.

We present our approach to applied learning on gender integration and analysis and reflect on inclusive approaches to training to identify remaining gaps and future directions. Our work at the Gender and Health Applied Learning Institute (i.e., the Gender and Health Summer Institute) aims to complement and expand these existing programs, focusing on global reach and enhanced accessibility, particularly via anti-oppressive pedagogy.

The Gender and Health Summer Institute (GHSI) (https://publichealth.jhu.edu/academics/summer-institute-in-gender-and-health) was launched in 2023 at the Johns Hopkins Bloomberg School of Public Health (BSPH). The institute currently offers 18 short courses focused on building applied skills in gender and health including gender analysis in health research and interventions, gender transformative programming, male engagement, gender responsive monitoring and evaluation, gender budgeting, adapting programs for gender minority individuals, communicating and advocating for gender-responsive science, and integrating gender into implementation research.

The institute and its courses were purposefully designed to reach a diverse, global audience. The courses are structured around Universal Design principles, which aim to make “teaching and learning products and environments usable by all people, to the greatest extent possible, without the need for adaptation or specialized design” (Burgstahler, 2009). Courses are open to everyone with the equivalent of a bachelor's degree. All courses in the institute are offered synchronously online from 8 AM to 12 PM Eastern Standard Time, to maximize participation from students around the world. Courses can be taken for credit or not-for credit, with the latter option offered at a significantly reduced rate, (~86% less per credit).

To address cost barriers, in 2024 we launched a scholarship program and received overwhelming interest with over 600 applications. All eligible applicants were reviewed by two GHSI faculty and given scores based on their expressed interest in the course, the perspectives and experiences they brought to the GHSI, and their proposed plan to apply the knowledge and skills gained through the course. Highest ranking applicants were then matched to their courses of interest and efforts were made to spread awardees across GHSI courses. We successfully awarded 88 scholarships, fully covering courses taken both for-credit and non-credit. The cost of each scholarship ranged from $400 to $4,122 USD.

## Actionable recommendations

We recommend the use of Universal Design principles in the design and implementation of applied gender training programs to enhance access and equity. A summary of these principles and their application within the GHSI are presented in Table 1. The design considerations can be taken up and tailored by other groups seeking to offer such training.

**Table 1:** Institute and course design considerations mapped to universal design principles.

**Universal design principle**	**Design considerations**
**Engagement:** For purposeful, motivated learners, stimulate interest and motivation for learning.	• Reduced tuition rates for non-credit participants and full scholarships for 95 enrollees in 2024. • Synchronous online sessions that are half day, starting in the morning EST.
**Representation:** For resourceful, knowledgeable learners, present information and content in different ways.	• Diverse perspectives: guest speakers, open-access materials for a broad array of sources. Targeted inclusion of non-Western voices, ways of thinking, knowing, and doing, and non-academic materials. • Synchronous and asynchronous learning modules. • Automatic live transcript and translated zoom captions into 39 languages.
**Action and expression:** For strategic, goal-directed learners, differentiate the ways that students can express what they know.	• Collaborative online platform for participants, instructors, and institute-affiliated staff. • Variety of co-learning approaches, including online participation tools, “flipped” classrooms, and interactive activities that encourage collaboration.

## Remaining gaps and future directions

Despite efforts to diversify participants and remain accessible to a global audience, most gender training institutes, including the GHSI, are currently located in and/or led by institutions based in HICs. This continues to pose a challenge for delivery, adaptability, and applicability to LMIC contexts. We acknowledge this as an important area of opportunity, particularly for institutions based in LMICs, to develop similar models of training.

## Conclusion

Gender integration and analysis is a rapidly growing discipline, spurred on by requirements of donor agencies and multi-lateral organizations. There is increasing recognition of the need for increased intervention upstream to tackle embedded and structured gender inequities which have historically disadvantaged women and girls. There is also recognition that lack of gender integration into health research and programming can lead to substantial negative unintended consequences—which can particularly harm women, girls, and gender minority individuals. Health research and interventions are implemented within complex social systems and structures which privilege certain groups of individuals over others; it is important to understand how these social systems structures operate and how they impact individual health and wellbeing. Health programs and interventions can no longer be implemented without considering individuals' lived experiences and gender integration and analysis is integral to this.

Globally, there is a need for better training on gender integration and analysis. Through our gender and health applied learning institute, we attempted to fill a substantial need for accessible and applied gender integration and analysis training. Our aim is to create a cadre of gender specialists embedded within professional organizations who can advocate for and implement more robust gender integration and analysis within their work, leading to more gender—responsive programming and reductions in health inequities globally. We hope that similar models will be developed and implemented globally.

## Data Availability

The original contributions presented in the study are included in the article/supplementary material, further inquiries can be directed to the corresponding author.
